# Experimental transmission of atypical scrapie to sheep

**DOI:** 10.1186/1746-6148-3-20

**Published:** 2007-08-28

**Authors:** Marion M Simmons, Timm Konold, Hugh A Simmons, Yvonne I Spencer, Richard Lockey, John Spiropoulos, Sharon Everitt, Derek Clifford

**Affiliations:** 1 Department of Pathology, Veterinary Laboratories Agency – Weybridge, Woodham Lane, New Haw, Addlestone, Surrey KT15 3NB, UK; 2 Department of Molecular Pathogenesis and Genetics, Veterinary Laboratories Agency – Weybridge, Woodham Lane, New Haw, Addlestone, Surrey KT15 3NB, UK; 3 Animal Services Unit, Veterinary Laboratories Agency – Weybridge, Woodham Lane, New Haw, Addlestone, Surrey KT15 3NB, UK

## Abstract

**Background:**

Active surveillance for transmissible spongiform encephalopathies in small ruminants has been an EU regulatory requirement since 2002. A number of European countries have subsequently reported cases of atypical scrapie, similar to previously published cases from Norway, which have pathological and molecular features distinct from classical scrapie. Most cases have occurred singly in flocks, associated with genotypes considered to be more resistant to classical disease. Experimental transmissibility of such isolates has been reported in certain ovinised transgenic mice, but has not previously been reported in the natural host. Information on the transmissibility of this agent is vital to ensuring that disease control measures are effective and proportionate.

**Results:**

This report presents the successful experimental transmission, in 378 days, of atypical scrapie to a recipient sheep of homologous genotype with preservation of the pathological and molecular characteristics of the donor. This isolate also transmitted to ovinised transgenic mice (Tg338) with a murine phenotype indistinguishable from that of Nor 98.

**Conclusion:**

This result strengthens the opinion that these cases result from a distinct strain of scrapie agent, which is potentially transmissible in the natural host under field conditions.

## Background

TSE in sheep have been recorded for more than 250 years in the form of classical scrapie. In classical scrapie, there is a clear link between susceptibility and sheep genotype [1], and many countries are now implementing disease control programmes based on breeding for resistance. Host genotype also appears to affect to some extent the ultimate pathological presentation of the disease [2-4] as do specific scrapie 'strains' [5] and this complex interaction is not yet clearly understood, although it is widely accepted that several variants of classical scrapie exist in the field. However, in 2003, a newly identified form of scrapie (Nor98) was reported in Norway, which was distinct from the established range of classical scrapie phenotypes [6].

In 2002, regulatory requirements for active TSE surveillance were implemented across the EU, and a number of countries have subsequently reported atypical isolates, full details of which can be found in the EFSA opinion of 2005 [7]. It has also recently been reported for the first time in the Southern hemisphere, in a sheep native to the Falkland Islands [8].

The current minimum scrapie control measures within the EU require selective culling within a flock, based on genotype susceptibility to classical scrapie. However, the susceptible genotype range for atypical cases differs from that of classical disease [7,9-11] with most cases occurring in genotypes associated with relative resistance to classical scrapie, and so other measures need to be considered in flocks where only atypical scrapie is identified. This is particularly pertinent given the epidemiology of atypical scrapie, with the majority of cases occurring as singletons within a flock when arguably the existing culling policies will achieve little additional control.

It has already been established that such atypical isolates can transmit to over-expressing ovinised transgenic mice (Tg338, [12]) but it is crucial to the formulation of control policies to establish whether or not this is a stable strain of TSE which can transmit within the natural host.

The route of natural transmission for TSE is still unclear, so the approach taken to establishing transmissibility of such an agent is generally performed in a number of steps. Initial species susceptibility is established by transmission via intracerebral inoculation, which optimises the delivery of the agent to the target organ, and minimises any reduction in efficiency of transmission resulting from a more peripheral route. Once such susceptibility is established, the outcome of challenges by more natural routes (e.g. oral challenge) is more informative.

This report presents the first successful intracerebral transmission of atypical scrapie within the natural host.

## Results

A 5 month old Cheviot sheep (with PrP genotype A_136_H_154_Q_171_/A_136_H_154_Q_171 _[1]) was inoculated intracerebrally with cerebellum from a case of atypical scrapie (5 year old Portland sheep, genotype AHQ/AHQ) identified through the fallen stock active surveillance programme. The recipient sheep was killed 378 dpi with signs of a neurological disease. These comprised behavioural changes (separation from others, confusion when the pen was entered, compulsive behaviour, such as nibbling movements of the lips (see Additional File 1) and circling clockwise (see Additional File 2), locomotor changes (mild ataxia with proprioceptive deficits) and loss of weight (16% in the preceding 6–8 weeks) and bodily condition. Pruritus was not a feature of this disease. The clinical findings were suggestive of a diffuse brain disease, which particularly involved the forebrain. The clockwise circling suggested an asymmetric lesion, but it was not possible to investigate this further, because only one half of the brain was fixed for histopathology. Behavioural changes were first reported by animal care staff at 314 dpi.

**Figure 1:**
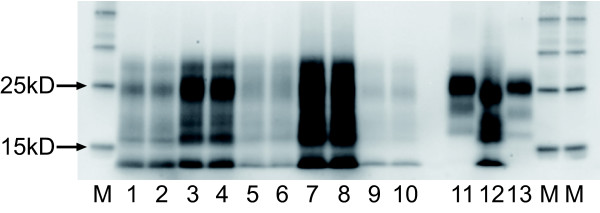
**BioRad Western Blot (10 min Detection)**. 15 μl of sample is loaded in each lane, giving a tissue equivalent of 0.02 g. Each sample is run in duplicate in adjacent lanes. Lanes 1 and 2 UK passive surveillance atypical scrapie (ARQ/AHQ; cortex). Lanes 3 and 4 UK passive surveillance atypical scrapie (AHQ/ARR; cortex). Lanes 5 and 6 UK active surveillance atypical scrapie (AHQ/ARR; medulla). Lanes 7 and 8 Donor sheep. UK active surveillance atypical scrapie (AHQ/AHQ; medulla). Lanes 9 and 10 Recipient sheep. Experimental atypical scrapie (AHQ/AHQ; medulla). Lane 11 UK classical scrapie positive control (VRQ/VRQ; medulla). Lane 12 Swedish Nor98 positive control (kindly supplied by Dr D Gavier-Widen, SVA, Sweden). Lane 13 UK bovine BSE positive active surveillance case control (brainstem). Lanes M Molecular mass markers.

A Western blot (Figure 1) prepared using the BioRad TeSeE™ revealed a banding pattern consistent with Nor98 [6], with a distinct low molecular mass band at approximately 12 kD. This pattern matched exactly the banding pattern of the donor sheep, and was consistent with the molecular characteristics of other UK atypical scrapie field isolates run on the same gel.

**Figure 2:**
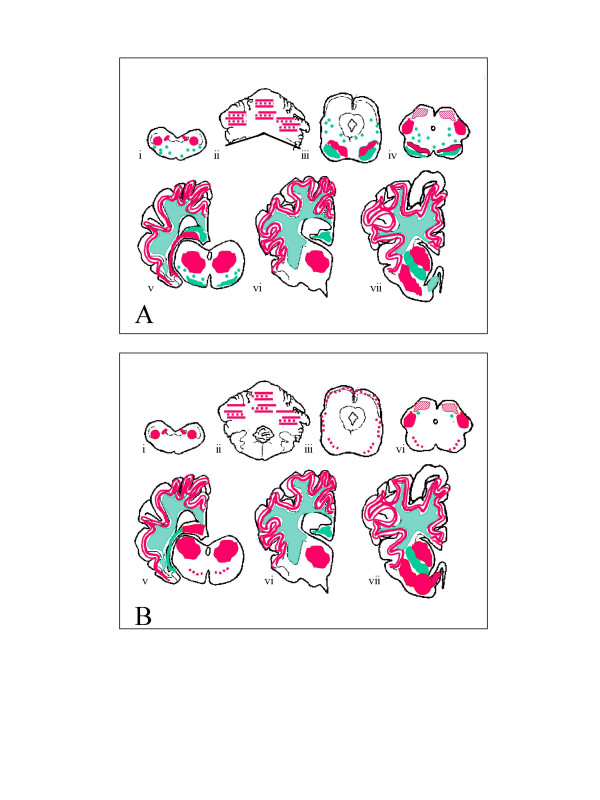
**Distribution and type of PrP immunolabelling in the donor (A) and the recipient sheep (B)**. Red = fine particulate staining of the neuropil. Green = coarse particulate/globular staining in the white matter. i) medulla oblongata (level of the obex); ii) rostral medulla/cerebellum; iii) caudal midbrain; iv) rostral midbrain; v) thalamus/occipital cortex/hippocampus; vi) rostral thalamus/hypothalamus/parietal cortex; vii) frontal cortex/basal ganglion.

Comparison of the distribution and type of PrP^Sc ^immunolabelling in the brains of both the donor and recipient animals revealed preservation of the immunopathological features following transmission (Figure 2). Immunolabelling in the medulla at the level of the obex was minimal, and confined to fine granular deposits in the nucleus of the spinal tract of the trigeminal nerve. Fine granular PrP^Sc ^was distributed widely throughout the grey matter of the cerebellar and cerebral cortices, and the neuropil of the basal ganglia and thalamus in both donor and recipient. Much less labelling was seen in the midbrain and medullary regions. Globular or semi-globular staining in the white matter, possibly associated with oligodendrocytes, was also widely distributed throughout the brain, again with relative sparing of the brainstem regions. There was no intracellular labelling seen in either case, which is consistent with observations in other atypical scrapie field cases, and different from classical scrapie (Simmons and Spiropoulos, personal observations) Apart from a small amount of vacuolation in the molecular layer of the cerebellum in the recipient animal, there was no TSE-associated vacuolation observed in either animal. No immunolabelling could be demonstrated in the LRS areas examined in the recipient animal (mesenteric lymph node, palatine tonsil, spleen, Peyer's patches of the distal ileum and nictitating membrane), which is in keeping with the limited observations in a similar range of LRS tissues which have been made in five animals naturally affected with atypical scrapie (MM Simmons, unpublished observations). However, this might also be partially attributable to the intracerebral route, since only very restricted LRS involvement was present in the classical scrapie positive control, which is contrary to observations in natural disease, and following oral challenge [13].

A second animal, also of the AHQ/AHQ genotype, which received an equivalent challenge with the same inoculum is still alive and healthy at more than 900 dpi (as at:June 2007).

Transmission of the source inoculum to Tg338 mice, within a separate project (J Spiropoulos and PC Griffiths, unpublished observations), was successful, with an incubation period of 210 ± 4.8 days and a histological lesion profile and PrP^Sc ^distribution pattern consistent with Nor98 and French discordant cases [12].

## Discussion

To our knowledge this is the first report of the successful experimental transmission of atypical scrapie within the natural host. The retention of the molecular and pathological characteristics of the scrapie agent following this experimental transmission supports the hypothesis that disease in the donor animal was caused by a stable strain of the TSE agent which has phenotypic characteristics distinct from those associated with classical scrapie. Although clinical signs were not recorded from the donor animal because it presented dead (fallen stock), the signs seen in the challenged animal were consistent with those reported for the small number of passive surveillance cases so far identified in the UK [14,15] and elsewhere [6,16], which were characterised by gait abnormalities, abnormal behaviour and a relative lack of pruritic signs.

The transmission characteristics of atypical scrapie from different countries in Tg338 mice [12] and emerging data from similar transmissions of the isolate in this report indicate that at least one strain of atypical scrapie compatible with/indistinguishable from Nor98 is widespread across Europe, despite its relatively recent identification.

Although it has now been demonstrated to be experimentally transmissible within and between species via the intracerebral route, there is little epidemiological evidence for ease of transmission of this strain under field conditions, where scrapie is most likely transmitted via the oral route. This raises the question of how it came to be so geographically widespread. One hypothesis is that this represents a spontaneous genetic disease in sheep similar to the familial forms of TSE in man (e.g. GSS, familial CJD and FFI etc.), in which the resultant disease can subsequently be transmitted experimentally [17,18].

Horizontal transmission of classical scrapie has been shown to occur in adult sheep exposed to an affected flock [19] and even in animals which have had contact only with a contaminated environment (GI Dexter and SJ Bellworthy, personal communication). The environmental persistence of the classical scrapie agent is also supported by epidemiological evidence from Icelandic repopulation studies [20,21]. However, the low molecular mass (<14 kD) band of PrP^Sc ^seen in Western blots of atypical isolates is associated with a reduction in PK resistance of this disease-associated protein. It is possible that this relative protease susceptibility may make the agent less robust in field conditions and thereby reduce the extent to which it is transmissible by natural routes, and so limit the number of cases. It could be argued that this in turn supports the hypothesis that the natural disease may arise spontaneously. However, although genetic studies have indicated a PrP genotype host range for atypical scrapie which, for the majority of cases, is almost fully distinct from that of the classical forms of disease, they have not identified a single genetic feature (such as the point mutations in some human forms [22]) which might account for this.

This relative PrP^Sc ^fragility [23] compared to the PrP^Sc ^in classical scrapie, may also account for the total absence of intracellular PrP^Sc ^immunolabelling. The different PK cleavage sites in PrP associated with different strains of TSE infecting small ruminants can be used to differentiate them, both in blots and by immunohistochemistry. Intracellular immunostaining is significantly reduced in sheep infected with BSE compared to classical scrapie, when the appropriate monoclonal antibodies are applied, and it has been speculated that this demonstrates a partial digestion of the PrP^Sc ^molecule by the endogenous proteases within the cells [24]. If the PrP^Sc ^is more protease sensitive, then this loss of intracellular immunolabelling even with an antibody to the core of the protein (such as MAb 2G11) might indicate that cells can more effectively catabolise this abnormal form of the protein than more 'classical' forms, and therefore succumb to clinical disease much later, if at all. This may also account for the apparent absence of disease-related PrP^Sc ^in lymphoid tissues. It is interesting to note that in familial CJD (E200K mutation) also, PrP labelling was seen as a uniformly fine deposit throughout all cortical layers [25].

This study confirms that 'atypical scrapie' is transmissible within the natural host species, via the intracerebral route, without alteration of the pathological and molecular characteristics. This is the first report from a larger study which will explore more widely the effect of the PrP gene on the experimental susceptibility and resultant phenotype following intracerebral or oral challenge with this type of scrapie isolate.

## Conclusion

At present the significance of this result, in terms of the transmissibility or pathogenicity under 'field conditions' of this agent strain in any species remains speculative, but it supports the need for appropriate control measures protecting both the animal and the human food chain to encompass atypical scrapie cases specifically.

## Methods

### Inoculum preparation and inoculation

This experimental case is part of a larger study involving oral as well as intracerebral routes of challenge. The phase of this study which involved intracerebral challenge is described here briefly for context. Four atypical isolates identified through active surveillance were used for this study. Two 'donor' animals were of the AHQ/AHQ genotype, and two were ARR/ARR. Material from each donor animal was prepared as a 10% homogenate in normal saline, checked for microbiological sterility and treated by antibiotic (ampicillin, or gentamycin (+/- heat if required)) prior to use. One ml of each homogenate was inoculated intracerebrally into two homologous and two 'classically susceptible' (ARQ/VRQ) recipient sheep, all derived from the VLA New Zealand-derived flock, and ranging from 5.5 to 11 months old at the time of inoculation.

All inoculations were carried out under general anaesthesia, and in accordance with the UK Animal (Scientific Procedures) Act 1986, under Licence from the UK Government Home Office (Project licence no: 70/5780). Such licence is only granted following approval by the internal VLA ethical review process as mandated by the Home Office.

One ARQ/VRQ classical scrapie case (into two ARQ/VRQ animals) was used as a positive control. Both recipient sheep succumbed to disease at 537 and 605 dpi respectively. A further positive control was provided by using a VRQ/VRQ classical scrapie case inoculated into a VRQ/VRQ recipient animal which developed clinical disease (pruritus, ataxia and weight loss) at 126 dpi. Brain from one ARR/ARR animal from the VLA New Zealand-derived flock was inoculated into an ARQ/VRQ recipient as a negative control. This animal died of intercurrent disease at 911 dpi, and was negative. All sheep are kept indoors and in fully segregated groups (based on inoculum received) and are alive and healthy, with the exception of two AHQ/AHQ animals which received AHQ/AHQ inoculum (a different source inoculum from the one used in the successful transmission reported in this paper), which are showing neurological abnormalities at approx 860 dpi.

### Clinical assessments

Animals were monitored daily during routine husbandry procedures (feeding, bedding) and weighed monthly. If clinical disease was suspected the animals were examined clinically and neurologically [26], which included testing of the scratch reflex to assess if a stereotypical response ('nibble reflex') could be elicited [15]. Prior to cull, animals were also monitored by CCTV for signs of pruritus (scratching, rubbing or nibbling body parts) or other behavioural abnormalities.

At clinical end-point, animals were killed using quinalbarbitone sodium (Somulose, Arnolds) and tissues collected into formal saline (for central nervous system tissues), neutral buffered formalin or stored frozen at -80°C. The brain was divided in half, with one half placed in fixative, and the other half stored frozen.

### Western Blotting

Fresh brain samples (either frontal cortex, or medulla) were subjected to the TeSeE sheep/goat Western blot (Bio-Rad Cat No: 355 1169). Tissue (0.35 g) from each sample was ribolysed, purified, PK treated and PrP^res ^concentrated following the kit instructions and reagents supplied. Samples were heated for 4 mins at 100°C prior to loading on gels. ECL DualVue (Amersham) molecular mass markers were included at either end of the gel. A single lane each of a UK classical ovine scrapie, UK bovine BSE and a Nor98 ovine scrapie case were included for profile comparisons. Fifteen microlitres of each sample (tissue equivalent of 0.02 g) was loaded in duplicate lanes onto pre-cast 12% bis-tris gels (Criterion) and electrophoresed for 50 mins at 200 V. The proteins were then transferred onto PVDF membranes (115 V for 60 min) and blocked (Bio-Rad blocking solution) for 40 mins at room temperature. They were probed with the kit primary antibody (monoclonal antibody SHA31, diluted to 1:10 of the supplied kit concentration) for 30 mins at room temperature. The membranes were washed, incubated for 20 mins in Bio-Rad secondary antibody at room temperature, washed again and the membranes were incubated with ECL substrate (Amersham) for 45 secs – 1 min. The signal was detected with the Fluor-S MultiImager (Bio-Rad).

### Immunohistochemistry

Immunohistochemical detection of PrP^Sc ^was performed using mouse Mab 2G11 (Institute Pourquier), raised against ovine PrP peptide sequence 146-R^154^R^171^-182.

Tissue sections were de-waxed and rehydrated routinely. Epitope demasking was performed by immersion of sections for 30 minutes in undiluted formic acid, then washed in running tap water for 15 minutes, followed by autoclaving at 121°C in citrate buffer pH 6.1 (8.8 mM tri-sodium citrate dihydrate, 1.3 mM citric acid in 2 litres purified water). Endogenous peroxidase was blocked using 3% hydrogen peroxide (100 vol) in methanol, and washing buffer used throughout the procedure was tris buffered saline, supplemented with 0.2% tween20 (TBST). Primary antibody was applied at dilution of 1/400 for one hour at room temperature, with immunodetection performed using biotinylated goat anti mouse and avidin-biotin-peroxidase-complex (Vector Elite) technique using diaminobenzidine chromogen prepared in McIlvane's citrate buffer. Sections were counterstained using Mayers haematoxylin, then routinely dehydrated, cleared and mounted in dibutylphthalate in xylene (DPX), before examination by light microscopy.

## Abbreviations

CCTV Closed circuit television

CJD Creutzfeldt-Jakob disease

dpi days post inoculation

DPX dibutylphthalate in xylene

ECL Enhanced chemiluminescence

EFSA European Food Safety Authority

EU European Union

FFI Fatal familial insomnia

GSS Gerstmann-Sträussler-Scheinker disease

LRS Lymphoreticular system

Mab Monoclonal antibody

PK proteinase K

PrP prion protein

PrP^res ^Proteinase K resistant, disease-associated prion protein

PrP^Sc ^PrP scrapie – the disease-associated isoform of prion protein

PVDF polyvinylidene fluoride

TSE transmissible spongiform encephalopathy

UK United Kingdom

## Authors' contributions

MMS designed and led the project, interpreted the pathology and drafted the manuscript; TK performed the neurological examinations, interpreted the clinical data and contributed to drafting the manuscript; HAS bred and supplied the scrapie-free recipient sheep, was responsible for the logistics of the live animal phase and helped to draft the manuscript; YIS was responsible for the optimisation of the immunohistochemical techniques, and contributed to the interpretation of the pathology and the drafting of the manuscript; RL undertook all the preparation of the inocula, was responsible for the quality assurance of all related data and contributed to the manuscript; JS contributed to the interpretation of the pathology and helped to draft the manuscript; SE performed and interpreted the Western blotting and contributed to the manuscript; DC undertook the surgical procedures and routine clinical monitoring, and helped to draft the manuscript.

All authors read and approved the final manuscript.

## Supplementary Material

Additional file 1 Lip movements. These spontaneous lip movements were not associated with other behaviour, such as rubbing of body parts. Click here for file

Additional file 2 Circling. This animal circles in a clockwise direction. Click here for file
